# Baseline left bundle branch block with right bundle branch escape complexes in a patient with coronary artery disease, presents like an alternating bundle branch block: a case report

**DOI:** 10.1186/1757-1626-1-427

**Published:** 2008-12-30

**Authors:** Arvind Bhimaraj, Salaheldin Abusin, Bosko Margeta

**Affiliations:** 1 Division of Cardiology, John. H. Stroger Hospital of Cook County, Chicago, IL, USA; 2 Department of Medicine; John. H. Stroger Hospital of Cook County, Chicago, IL, USA

## Abstract

Alternating bundle branch block (ABBB) is a less commonly encountered phenomenon with the advent of re-perfusion therapy for acute myocardial infarction. ECGs simulating the appearance of an ABBB need to be carefully analysed. We present an ECG showing a baseline Left Bundle Branch Block(LBBB) progressing to a high grade AV block with escape complexes having a Right Bundle Branch Block (RBBB) morphology. Such an ECG can be mistaken for an ABBB if not analysed carefully.

## Introduction

Alternating Bundle Branch Block (ABBB) is when both right bundle branch block (RBBB) and left bundle branch block (LBBB) patterns appear on the same ECG or within a period of hours to days [1]. Before the re-perfusion era, it occurred in up to 6% of all forms of bundle branch block [2] following acute myocardial infarction, with 44% progression rate to high grade AV block [3]. With the advent of modern re-perfusion therapy it is now less commonly encountered in clinical practice. Rhythms that simulate an ABBB need to be analysed closely. Such rare ECG patterns with fusion and escape complexes simulating an ABBB have been described earlier [4]. We present an interesting ECG simulating an ABBB.

## Case presentation

A 77 year old female presented to our institution with the chief complaint of severe substernal chest pressure that occurred at rest, sudden in onset, initially severe with a decrease in intensity in 30 minutes and completely resolved after receiving sublingual nitroglycerin in the ER. She reported three weeks history of exertional chest pain and exertional dizziness for 6 months. Her medical history was significant for Diabetes Mellitus and Hypertension. She had a significant family history of CAD with her son being diagnosed at the age of 40. Her medications included insulin, ezetimibe-simvastatin and aspirin. Her examination was significant for a heart rate of 35 beats/min, blood pressure of 168/72 and mild pedal edema.

**Figure 1:**
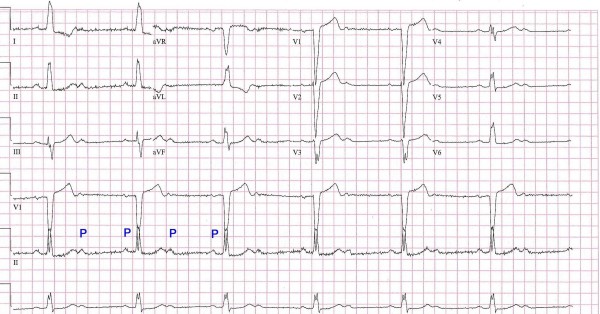
**Second degree heart block with 2:1 conduction and LBBB**.

The first ECG on admission (Figure 1), showed a sinus rate of 75 with a 2:1 conduction and LBBB.

**Figure 2:**
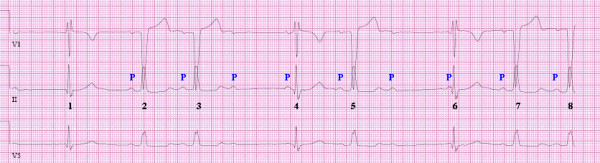
**LBBB with 2^nd ^degree AV block with complexes of RBBB morphology-(probably escape rhythm)**.

A second ECG 2 hours later (Figure 2) showed bradycardia, the 2^nd^, 3^rd ^5^th ^7^th ^and 8^th ^QRS complexes had LBBB morphology with first degree AV block conduction (PR interval of 0.208 seconds). The 1^st^, 4^th ^and 6^th ^QRS complexes showed RBBB morphology with what initally appears as 2^nd ^degree type II block in the 4^th ^and 6^th ^complexes. On superficial evaluation, the ECGs suggest the presence of an alternating bundle branch block, but on a closer look it is seen that the PR intervals of the RBBB morphology complexes are too short making us suspect complete AV conduction block. Furthermore, the first QRS complex in figure 2 shows no P waves. As a result QRS complexes of RBBB morphology are probably escape beats.

Her laboratory investigations including cardiac enzymes were unremarkable. In view of her chief complaint, she underwent a coronary angiogram that showed a 80% mid and a 70% distal RCA stenosis and 60% stenosis in the mid segment of the first diagonal branch of the LAD. Percutaneous Coronary Intervention (PCI) of the RCA was performed with good angiographic results. Her echocardiogram showed preserved systolic function, and grade 1 diastolic dysfunction with normal valves.

Following angioplasty, her conduction abnormality persisted with type II second degree AV block and a pacemaker was implanted because of the presence of a symptomatic conduction abnormality.

At six months follow up she was doing well with no episodes of syncope or chest pain.

## Discussion

Bundle branch blocks can occur as a part of a diffuse conduction system disease, myocardial disease or focal disruption due to ischemia/infarction. Due to the extent of branching of the left bundle, LBBB more often is associated with either diffuse disease pattern or proximal ischemia in the left anterior descending territory. The occurrence of an escape rhythm with RBBB morphology, in the presence of a LBBB suggests that the focus of origin of the latter is in the left bundle, distal to the conduction block. It is not clear if the presence of such an automaticity in the left bundle suggests the cause of the baseline LBBB in our patient to have occurred due to a focal disruption of the left bundle (due to ischemia/infarction)rather than a diffuse degenerative process (which was the presumed cause of the baseline LBBB in our patient). The only report we could find similar to ours was by Gimbell in 1972 in which such an ECG was portrayed as a surrogate of alternating bundle branch block. Our patient did not receive any electrophysiological studies due to personal choice but a better understanding would have been possible by doing so.

## Conclusion

The significance of an escape rhythm with an opposite bundle branch morphology compared to a baseline conduction abnormality is unknown. Close attention should be paid to ECGs which look like Alternating Bundle Branch Block in order to better study such phenomenon.

## Consent

Written informed consent was obtained from the patient for publication of this case report and accompanying images. A copy of the written consent is available for review by the Editor-in-Chief of this journal.

## Competing interests

The authors declare that they have no competing interests.

## Authors' contributions

AB and SA analyzed and interpreted the patient data and were major contributors in writing the manuscript. BM was the cardiology attending in charge of the patient and was a major contributor in writing the manuscript. All authors read and approved the final manuscript.
